# Mathematical modelling to determine the greatest height of trees

**DOI:** 10.1038/s41598-022-06041-w

**Published:** 2022-02-07

**Authors:** Tohya Kanahama, Motohiro Sato

**Affiliations:** 1 Graduate School of Engineering, Hokkaido University, Kita 13, Nishi 8, Kita-ku, Sapporo, 060-8628 Japan; 2 Faculty of Engineering, Hokkaido University, Kita 13, Nishi 8, Kita-ku, Sapporo, 060-8628 Japan

**Keywords:** Engineering, Civil engineering, Mechanical engineering

## Abstract

This study aimed to analyse the critical height of a column whose weight varies vertically in order to obtain a simple scaling law for a tree where the weight distribution considered. We modelled trees as cantilevers that were fixed to the ground and formulated a self-buckling problem for various weight distributions. A formula for calculating the critical height was derived in a simple form that did not include special functions. We obtained a theoretical clarification of the effect of the weight distribution of heavy columns on the buckling behaviour. A widely applicable scaling law for trees was obtained. We found that an actual tree manages to distribute the weight of its trunk and branches along its vertical extent in a manner that adequately secures its critical height. The method and findings of this study are applicable to a wide range of fields, such as the simplification of complicated buckling problems and the study of tree shape quantification.

## Introduction

Over the course of evolution, living organisms have acquired a remarkable degree of wisdom. For example, standing plants in nature have acquired mechanisms to efficiently resist various external forces, such as bending, shear, and buckling^1,2^. Many previous studies have focused on the bamboo, which has a peculiar structure, investigating the rationality of this structure^3,4^. A wild bamboo has an extremely long and slender body. The shape as such possesses a poor resistance to buckling. However, previous studies found that the bamboo reduces self-weight by incorporating a hollow structure, such that its buckling resistance is effectively improved by adjusting the node interval and the vascular bundle distribution.

Based on those prior results, this study focused on trees, which are self-standing plants similar to bamboo. The bamboo consists of a hollow cylinder with light branches and leaves. In contrast, trees have a solid cross-section and a heavy body. Therefore, the buckling risk under self-weight highly increases as trees grow taller. Nevertheless, trees in the wild naturally acquire appropriate heights and concomitant levels of mechanical stability that are adapted to harsh natural environments. This implies that mechanical strategies for avoiding self-buckling are incorporated in the forms adopted by trees.

Greenhill^5^ conducted a study on the self-buckling of trees. He modelled a tree as a cylindrical cantilever and derived the exact solution for the critical height for self-buckling as follows:

1 $${l}_{c}={\left(C\frac{E}{\gamma } {r}^{2}\right)}^{1/3}$$

where $E$ is the modulus of elasticity $\left[\text{N/}{\text{m}}^{2}\right]$, $\gamma$ is the unit volume weight $\left[\text{N/}{\text{m}}^{3}\right]$, $r$ is the radius $\left[{\text{m}}\right]$, and $C$ is a constant ($C\approx \text{1.959}$). Equation (1) shows that the critical height of a tree is proportional to the 2/3 power of the radius. McMahon proved that this law is correct for wild trees^6^. This scaling law has been applied widely in forest science and ecology^7–10^ owing to its simplicity. von Karman and Biot^11^ solved the governing differential equation by using a series solution and derived a formula for the critical height, which is almost equivalent to Greenhill's equation ($C\approx \text{2.0}$).

Other factors that determine critical height are hydraulic conditions^12,13^, wind force effects^14,15^, and genetic factors^16^. The scaling laws found by Greenhill correspond to the findings of McMahon^6^, which indicated that the equation formulating the critical height for self-buckling includes scaling laws that are widely applicable to trees. Most of the prior studies of the scaling laws associated with trees considered that the critical height is determined by the self-buckling condition^17–19^.

Greenhill's formula is widely applied in the fields of forestry science and ecology, and it is also the starting point for research on self-weight buckling in civil and mechanical engineering. Grishcoff^20^ investigated a column that is loaded simultaneously with its self-weight and concentrated loads. Wang and Drachman^21^ extended Grishcoff's study to an inverted column. Whereas these solutions were approximate, Chai and Wang^22^ and Duan^23^ used different methods to obtain the exact buckling stresses under self-weight and concentrated loads. Zorica^24^ extended this problem to a diagonal cantilever beam.

The self-buckling problem of columns has been extended to tapered columns, which have recently been actively used in civil engineering structures^25^. A study of tapered columns was initiated by Smith^26^. He formulated the buckling problem for linearly tapered columns, which ignores the self-weight. Subsequent studies extended the problem scope to heavy columns with arbitrary taper ratios, using various approaches^27–29^. An investigation of curvilinearly-tapered columns^30^, another investigation that introduces a model that includes concentrated loads at arbitrary positions^31^, theoretical consideration regarding post-buckling behaviours of heavy columns^32–34^, and studies employing other considerations^35,36^ have been performed. However, these studies are highly complicated, and none of them expresses a simple law such as Greenhill's formula.

Previously, we theoretically derived a simple scaling law for a heavy tapered column for the first time^37^ with the aim of applications in engineering as well as in ecology and forest science. We found that the critical height of a linearly tapered column is inversely proportional to the 1/6 power of the ratio of the radius of the free end to the radius of the fixed end. We also found that the Greenhill power law holds even in a tapered column.

As aforementioned, the critical buckling height of trees and the self-buckling of columns have been investigated for various purposes and for various fields of application. However, the effects of branches and leaves, which are considered important factors for buckling resistance, were not considered in previous studies. A clarification of the relationship between the critical height and the weight and distribution of branches and leaves will have significant engineering implications, such as how to distribute weight in the design of tower structures and how to control or reduce the occurrence of buckling under self-weight. Furthermore, a simple scaling law for the buckling length and the leaf weight of trees, similar to Greenhill’s equation, would be of great value in forestry science, ecology, and other fields of research.

The purpose of this study is to derive the critical height of a column whose weight varies in the vertical direction. We modelled the trees as cantilevers that were fixed to the ground. We formulated a self-buckling problem that took account of various weight distributions by using the series solutions of von Karman and Biot^11^. In considering real trees, the weight distribution of the trunk of a tree was assumed to be constant, and the branches were assumed to be variable quantities in the vertical direction. The formula for the critical height was derived in a simple form that did not include special functions, similar to the Greenhill formula. The effect of the weight distribution of heavy columns on the buckling behaviour was theoretically clarified, and a widely applicable scaling law was obtained.

The methods and results presented herein provide the buckling length of a heavy column considering its weight distribution. They also simplify the treatment of conventional complex buckling problems in engineering^29,30^. Furthermore, they can be used to investigate the rationality of tree morphology, to study tree shape quantification in forest science^38,39^, as well as in a wide range of other applications.

## Methods

### Density function

**Figure 1:**
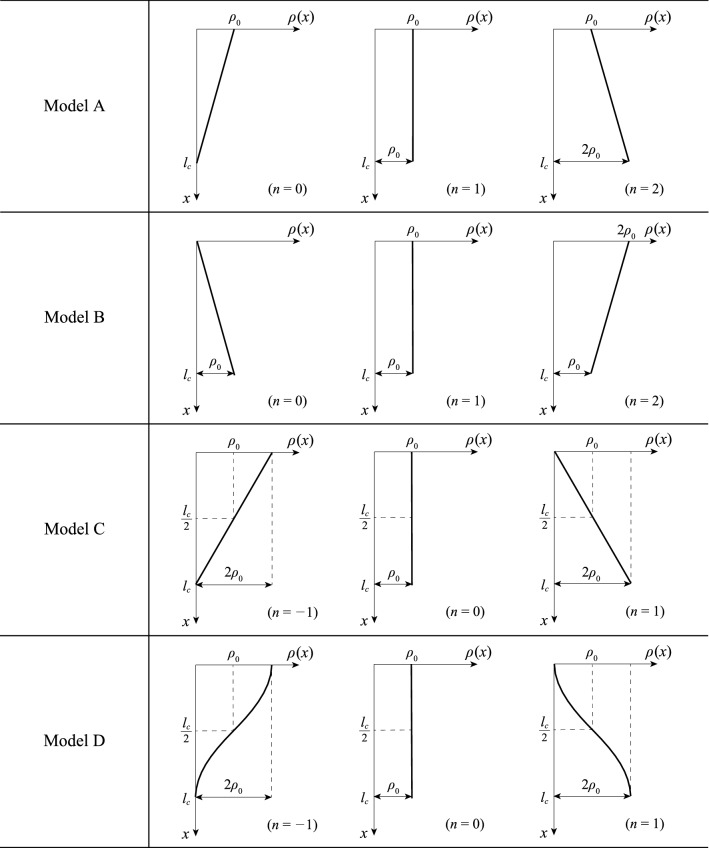
Density functions for models A, B, C, and D, respectively.

In previous studies, the density of a tree was treated as constant in the vertical direction ($\rho \left(x\right)=const.$). However, in this study, the density was treated as a function of height to clarify the relationship between the weight distribution and the critical height of a tree. We considered the following density functions:

2 $${\text{Model A}}{\text{: }}\rho \left(x\right)=\left(\frac{n-1}{{l}_{c}}x+1\right){\rho }_{0 }{\text{,}}$$

3 $${\text{Model B}}{\text{: }}\rho \left(x\right)=\left(\frac{1-n}{{l}_{c}}x+n\right){\rho }_{0} {\text{,}}$$

4 $${\text{Model C}}{\text{: }}\rho \left(x\right)=\left(\frac{2n}{{l}_{c}}x+\left(1-n\right)\right){\rho }_{0} {\text{,}}$$

5 $${\text{Model D}}{\text{: }}\rho \left(x\right)=\left(1-n\mathrm{cos}\left(\frac{\pi }{{l}_{c}}x\right)\right){\rho }_{0} {\text{,}}$$

where ${\rho }_{0}$ is the density at the reference point ${\text{[kg/}}{\text{m}}^{3}{\text{]}}$ and $n$ is a parameter that controls the shape of the density distribution. An example of the distribution of each model is shown in Fig. 1. The coordinate axis was aligned with the neutral axis, with $x=0$ at the free end and $x={l}_{c}$ at the fixed end.

Model A is a model in which the density at the free end is constant and independent of parameter $n$ ($\rho \left(0\right)={\rho }_{0}$) whereas the density at the fixed end is $n$ times the density at the free end ($\rho \left({l}_{c}\right)=n{\rho }_{0}$) . Model B in Eq. (3) is the inverted form of Model A, i.e., the density at the fixed end is constant $(\rho \left({l}_{c}\right)={\rho }_{0}$) whereas the density at the free end is $n$ times the density at the fixed end ($\rho \left(0\right)=n{\rho }_{0}$). For the same value of $n$ in Models A and B, both models have the same total weight. Based on the observation that the total weight of the branches rarely exceeds the total weight of the trunk^40,41^, we assumed that the range of $n$ in Models A and B was $0\le n\le 3$. Models C and D in Eqs. (4) and (5) are models in which the total weight of the system is independent of the parameter $n$ if the height is invariant. The difference between Models C and D is whether the distribution is linear or curved. In these models, the density distribution is constant in the vertical direction, as in Greenhill’s equation with $n=0$. Moreover, the weight is concentrated at the top when $n=-1$ and at the bottom when $n=1$. A summary of these results appears in Table 1. Not only to clarify the effect of weight distribution on the critical height, but also to ensure future expandability, we used the above four density distribution functions as they are geometrically simple and can represent various distribution shapes.

**Table 1:** Calculation models and related numerical conditions.

Model	Function	Range of $n$	Constant-density state
A	$\rho \left(x\right)=\left(\frac{n-1}{{l}_{c}}x+1\right){\rho }_{0}$	$0\le n\le 3$	$n=1$
B	$\rho \left(x\right)=\left(\frac{1-n}{{l}_{c}}x+n\right){\rho }_{0}$	$0\le n\le 3$	$n=1$
C	$\rho \left(x\right)=\left(\frac{2n}{{l}_{c}}x+\left(1-n\right)\right){\rho }_{0}$	$-1\le n\le 1$	$n=0$
D	$\rho \left(x\right)=\left(1-n\mathrm{cos}\left(\frac{\pi }{{l}_{c}}x\right)\right){\rho }_{0}$	$-1\le n\le 1$	$n=0$

**Figure 2:**
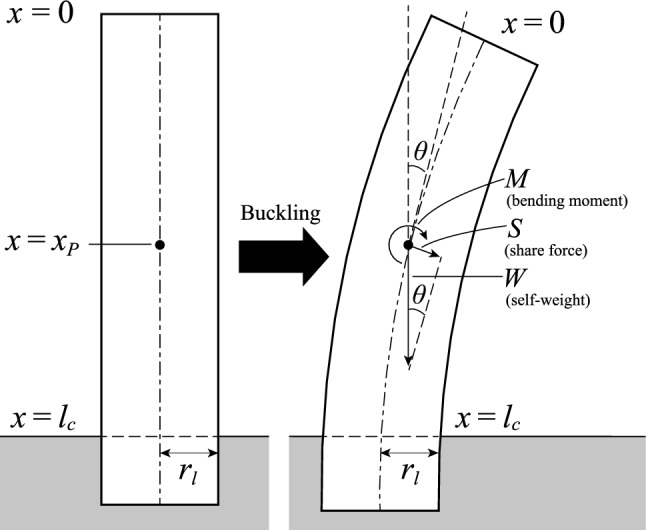
Calculation model.

The calculation model was a cantilevered elastic column with a radius ${r}_{l}\text{ [m]}$ and a length $l\text{ [m]}$ **(**see Fig. 2**)**. Greenhill's formula^5^ that was verified by McMahon^6^ and has been broadly used in various research, it had been derived by modelling trees as non-tapered cylinders. Furthermore, the geometrically simple models are desirable to obtain the wide applicability and extensibility. For the above reasons, we modelled trees as non-taper cylinders. The weight $W(x)$ from the upper end to any point $x$ is given by:

6 $$W=\iiint \rho \left(x\right)g \, dx \, dy \, dz={\int }_{0}^{x}\rho \left(x\right) Ag \, dx\text{,}$$

where $A$ is the cross-sectional area $\text{[}{\text{m}}^{2}\text{]}$. When the column buckles under its self-weight, the shear force $S\left(x\right)$ at any point $x$ is given by:

7 $$S\left(x\right) = W\left(x\right)\mathrm{sin}\theta ={\int }_{0}^{x}\rho \left(x\right) Ag \, dx\,\,\mathrm{sin}\,\theta \text{,}$$

where $\theta$ is the deflection angle. Note that the deflection angle $\theta$ is not included in the integration of Eq. (6) with respect to $x$ because this is only a volume integration to determine the weight $W(x)$. If we assume that $\theta$ is very small, $\mathrm{sin}\theta$ can be approximated as $\theta$. Therefore, Eq. (7) can be written as

8 $$S\left(x\right) \approx {\int }_{0}^{x}\rho \left(x\right) Ag \, dx\,\theta \text{,}$$

From the elastic curve equation of the beam, the bending moment $M\left(x\right)$ at any point $x$ can be obtained as

9 $$M\left(x\right)=-EI\frac{{d}^{2}y}{d{x}^{2}} \text{,}$$

where $y$ is the deflection. Because the deflection angle $\theta$ is very small, Eq. (9) can be approximated as:

10 $$M\left( x \right) \approx - EI\frac{{d\theta }}{{dx}}.$$

### Governing differential equation

From the relationship between the shearing force $S(x)$ of Eq. (7) and the end bending moment $M(x)$ of Eq. (10), the governing differential equation is obtained as

11 $$\frac{{d}^{2}\theta }{d{x}^{2}}+\frac{1}{EI}{\int }_{0}^{x}\rho \left(x\right)\, Ag \, dx\,\theta =0 \text{.}$$

Equation (11) is a second-order ordinary differential equation that has an independent variable $x$ and dependent variable $\theta (x)$. We integrated the equations that are obtained by substituting the density functions in Eqs. (2)–(5) for $\rho (x)$ in Eq. (11) and then applied the following transformation:

12 $$\xi \left(x\right)=\omega x \text{,}$$

where $\omega$ is a constant.

The governing differential equations were obtained in the following form:

13 $${\text{Model A: }}\frac{{d}^{2}\theta }{d{\xi }^{2}}+\left(\frac{n-1}{2{l}_{c}}\frac{\xi }{\omega }+1\right)\xi \theta =0 {\text{,}}$$

14 $${\text{Model B: }}\frac{{d}^{2}\theta }{d{\xi }^{2}}+\left(\frac{1-n}{2{l}_{c}}\frac{\xi }{\omega }+n\right)\xi \theta =0 {\text{,}}$$

15 $${\text{Model C: }}\frac{{d}^{2}\theta }{d{\xi }^{2}}+\left(\frac{n}{{l}_{c}}\frac{\xi }{\omega }+\left(1-n\right)\right)\xi \theta =0 {\text{,}}$$

16 $${\text{Model D}}{\text{: }}\frac{{d}^{2}\theta }{d{\xi }^{2}}+\left(1-\frac{{l}_{c}}{\pi }\frac{\omega }{\xi }\mathrm{sin}\left(\frac{\pi }{{l}_{c}}\frac{\xi }{\omega }\right)\right)\xi \theta =0 {\text{.}}$$

If we consider the governing equations in their simplest form, the constant $\omega$ for all the models can be defined as

17 $$\omega ={\left(\frac{{\gamma }_{0}A}{EI}\right)}^{1/3}{=\left(\frac{{\rho }_{0}gA}{EI}\right)}^{1/3}.$$

### Critical height formula

The general solution of the governing equations was obtained by the series solution method using Mathematica. For example, in Model C, the series solution of Eq. (15) is obtained as follows:

18 $$\theta \left(\xi \right)=\left(1-\frac{1}{6}{\xi }^{3}\left(n-1\right)+\frac{1}{180}{\xi }^{6}{\left(n-1\right)}^{2}+\cdots \right){c}_{1}+\left(\xi -\frac{1}{12}{\xi }^{4}\left(n-1\right)+\frac{1}{504}{\xi }^{7}{\left(n-1\right)}^{2}+\cdots \right){c}_{2}$$

where ${c}_{1}$ and ${c}_{2}$ are arbitrary constants.

We applied the following boundary conditions of the cantilever to the general solution of the governing differential equations, deriving the following algebraic equation for the critical height:

19 $$\left\{\begin{array}{c}\frac{d\theta }{d\xi }=0 \left(at ~ x=0\right)\\ \theta =0 \left(at ~ x={l}_{c}\right) \text{.}\end{array}\right.$$

By applying the boundary condition at the free end, we found that ${c}_{2}=0$ in Eq. (18). Moreover, by applying the boundary condition in the fixed end, Eq. (18) can be rewritten as

20 $$\left(1-\frac{1}{6}{\xi }^{3}\left(n-1\right)+\frac{1}{180}{\xi }^{6}{\left(n-1\right)}^{2}+\cdots \right){c}_{1}=0 .$$

By considering the condition for finding a non-trivial solution (${c}_{1}\ne 0$), we obtained the following algebraic equation with respect to $\xi$:

21 $$\left(1-\frac{1}{6}{\xi }^{3}\left(n-1\right)+\frac{1}{180}{\xi }^{6}{\left(n-1\right)}^{2}+\cdots \right)=0 .$$

When $n=0$, Eq. (21) corresponds to the equation of von Karman and Biot^11^. The critical height ${l}_{c}$ can be determined for any value of $n$ by calculating the smallest positive real number ${\xi }_{c}$ that satisfies Eq. (21) and substituting that value of ${\xi }_{c}$ into the following equation (which follows from Eqs. (11), (15), and (17)):

22 $${l}_{c}{=\frac{{\xi }_{c}(n)}{{\left(4C\right)}^{1/3}}\left(C\frac{E}{\gamma }{r}_{l}^{2}\right)}^{1/3}\text{,}$$

where $C$ is Greenhill’s constant ($C\approx 1.959$). Equation (22) is expressed in the form of the product of the coefficient varying with the density distribution and the critical height in the constant-density column introduce by Greenhill. Equation (21) has only two variables, ${\xi }_{c}$ and $n$. In other words, ${\xi }_{c}$ varies only with $n$. This property is the same for all density models, and the critical height can be obtained using the same method.

### Buckling mode

In this section, we describe the calculation of the buckling modes. First, we integrated Eq. (18) with respect to $\xi$, in order to obtain the deflection $y(\xi )$:

23 $$y\left(\xi \right)=\left(\xi -\frac{1}{24}{\xi }^{4}\left(n-1\right)+\frac{1}{1260}{\xi }^{7}{\left(n-1\right)}^{2}+\cdots \right){c}_{1}+{c}_{3} \text{,}$$

where ${c}_{3}$ is an arbitrary constant. By considering the boundary condition at the fixed end ($y\left(\omega {l}_{c}\right)=0$), ${c}_{3}$ is given by:

24 $${c}_{3}=-\left(\omega {l}_{c}-\frac{1}{24}{\left(\omega {l}_{c}\right)}^{4}\left(n-1\right)+\frac{1}{1260}{\left(\omega {l}_{c}\right)}^{7}{\left(n-1\right)}^{2}+\cdots \right){c}_{1 }\text{.}$$

Next, we substituted Eq. (24) into Eq. (23) and applied the condition $\xi =\omega {l}_{c}$, The deflection $y\left(x\right)$ was obtained as follows:

25 $$y\left(x\right)=\left(\omega \left(x-{l}_{c}\right) -\frac{{\omega }^{4}}{24}\left(n-1\right)\left({x}^{4}-{l}_{c}^{4}\right) +\frac{{\omega }^{7}}{1260}{\left(n-1\right)}^{2}\left({x}^{7}-{l}_{c}^{7}\right)+\cdots \right){c}_{1}\text{ .}$$

Because Eq. (25) includes the unknown coefficient ${c}_{1},$ we used the deflection ratio $y(x)/{y}_{max}$ to eliminate it. The maximum deflection ${y}_{max}$ appears at $x=0$, and the deflection ratio $y/{y}_{max}$ to draw the buckling modes is given by

26 $$\frac{y}{{y}_{max}}=\frac{\left(\omega \left(x-{l}_{c}\right) -\frac{{\omega }^{4}}{24}\left(n-1\right)\left({x}^{4}-{l}_{c}^{4}\right) +\frac{{\omega }^{7}}{1260}{\left(n-1\right)}^{2}\left({x}^{7}-{l}_{c}^{7}\right)+\cdots \right)}{\left(\omega \left(-{l}_{c}\right) -\frac{{\omega }^{4}}{24}\left(n-1\right)\left(-{l}_{c}^{4}\right) +\frac{{\omega }^{7}}{1260}{\left(n-1\right)}^{2}\left(-{l}_{c}^{7}\right)+\cdots \right)} \text{.}$$

Therefore, by substituting ${l}_{c}$ into Eq. (26), the buckling modes under self-weight could be obtained. We obtained the buckling modes for other density models with the same method.

### Example: application to real trees

**Figure 3:**
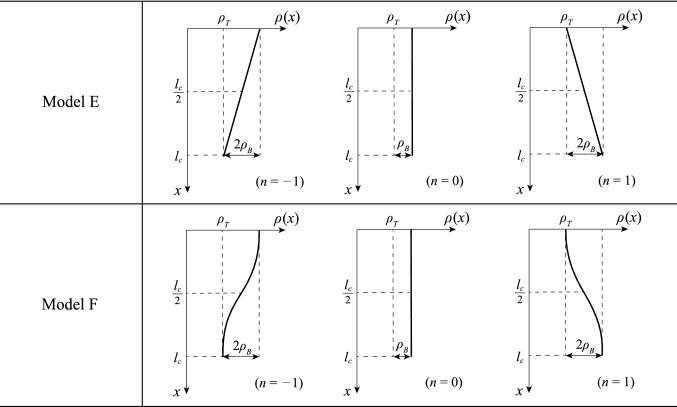
Examples of density distributions representing real trees.

In this section, we consider the effect of weight distribution on critical height in real trees by using the critical height formula derived by the aforementioned methods. Based on the investigation on the weight distribution of real trees^40,41^, we used the following functions to add the trunk weight to Models C and D, respectively:

27 $$\text{Model E: }\rho \left(x\right)=\left(\frac{2n}{{l}_{c}}x+\left(1-n\right)\right){\rho }_{B}+{\rho }_{T} \text{,}$$

28 $$\text{Model F: }\rho \left(x\right)=\left(1-n\mathrm{cos}\left(\frac{\pi x}{{l}_{c}}\right)\right){\rho }_{B}+{\rho }_{T} \text{,}$$

where ${\rho }_{B}$ is the density of the branches [kg/m^3^], and ${\rho }_{T}$ is the density of the trunk [kg/m^3^]. An example of the distributions in Eqs. (27) and (28) is shown in Fig. 3. In this study, we expressed the density ratio of the branches and the trunk, ${\rho }_{B}/{\rho }_{T}$, as a weight ratio ${W}_{R}$ because the density ratio of the branches and trunk is equal to their weight ratio. By using the same method as for the other models, the governing differential equations were obtained as follows:

29 $${\text{Model E}}{\text{: }}\frac{{d}^{2}\theta }{d{\xi }^{2}}+\left(\left(\frac{n}{{l}_{c}}\frac{\xi }{\omega }+(1-n)\right){W}_{R}+1\right)\xi \theta =0,$$

30 $${\text{Model F}}{\text{: }}\frac{{d}^{2}\theta }{d{\xi }^{2}}+\left(\left(1-\frac{{l}_{c}}{\pi }\frac{\omega }{\xi }\mathrm{sin}\left(\frac{\pi }{{l}_{c}}\frac{\xi }{\omega }\right)\right){W}_{R}+1\right) \xi \theta =0.$$

### Calculation

#### Method for solving the critical height equation

Algebraic solving of high-order equations such as Eq. (21) is impossible. Therefore, we used numerical computation to find the smallest positive real-valued solution that satisfies the high-order equation presented in this study. For the numerical method, we adopted the secant method, which does not require differentiation to allow for future extensions.

We rewrote Eq. (18) as follows:

31 $$\left(1-\frac{1}{6}{\xi }^{3}\left(n-1\right)+\frac{1}{180}{\xi }^{6}{\left(n-1\right)}^{2}+\cdots \right)=f\left(\xi \right) .$$

By increasing the value of $\xi$, we explored the interval $\left[{\xi }_{0},{\xi }_{1}\right]$ where $f\left({\xi }_{0}\right)\bullet f\left({\xi }_{1}\right)<0$. Using these values of ${\xi }_{0}$ and ${\xi }_{1}$ as initial values, we iterated using the following equation:

32 $${\xi }_{(k+1)}={\xi }_{(k)}-f\left({\xi }_{\left(k\right)}\right)\frac{{\xi }_{\left(k\right)}-{\xi }_{\left(k-1\right)}}{f\left({\xi }_{\left(k\right)}\right)-f\left({\xi }_{\left(k-1\right)}\right)}~~\left(k=1, 2,\cdots \right).$$

We used the following equation as a convergence criterion:

33 $$\left|\frac{{\xi }_{\left(k\right)}-{\xi }_{\left(k-1\right)}}{{\xi }_{\left(k\right)}}\right|\le \epsilon =1.0\times {10}^{-5} \text{.}$$

#### Convergence of series solutions

To determine the expansion order $N$ of the series solution in the high-order equation, we defined the convergence judgement function as follows:

34 $${f}_{(N,N+\Delta N)}=\left|\frac{{\xi }_{(N)}-{\xi }_{(N+\Delta N)}}{{\xi }_{(N+\Delta N)}}\right|\text{,}$$

where $\Delta N$ is the increment of the expansion order and ${\xi }_{\left(N\right)}$ is the minimum positive real solution in the high-order equation (Eq. (18)) when the expansion order is $N$. In many studies, the height of a tree is measured in centimetres^42,43^. Therefore, we used the following judgement function to determine the expansion order:

35 $${f}_{(N,N+\Delta N)}\le 1.0\times {10}^{-4} .$$

We increased $N$ by $\Delta N$ until the above condition was satisfied. The smallest expansion order $N$ that satisfied Eq. (35) was the expansion order used in this study.

#### Relationship between critical height and parameter $n$

In this section, we clarify the relationship between the critical height ${l}_{c}$ and the density distribution parameter $n$. The critical height ratio $f(n)$ was defined as follows:

36 $$f\left(n\right)=\frac{{l}_{c\left(n\right)}}{{l}_{cs}} \text{,}$$

where ${l}_{c(n)}$ was the critical height in the variable density model, and ${l}_{cs}$ was the critical height in the model with constant density. If the specification of each model is the same, the critical height is larger than the critical height of the density constant state when $f\left(n\right)$ is larger than 1. In contrast, the critical height is smaller than the critical height of the state with constant density when $f\left(n\right)$ is smaller than 1. Because ${l}_{c\left(n\right)}$ and ${l}_{cs}$ are given by Eqs. (22) and (1), respectively, the critical height ratio $f\left(n\right)$ was obtained as follows:

37 $$f\left(n\right)=\frac{{\xi }_{c}(n)}{{\left(4C\right)}^{1/3} }\text{ .}$$

In order to clarify the relationship between the critical height ratio $f\left(n\right)$ and the density distribution parameter $n$, we obtained the regression curve (discretely calculated) from $f\left(n\right)$. By using this method, the critical height ratio $f\left(n\right)$ was expressed as a simple function of $n$. By applying it to Eq. (22), the relationship between the critical height ${l}_{c}$ and the weight distribution was clarified. In Sect. 2.5, the critical height ratio $f\left(n,{W}_{R}\right)$ was defined as follows:

38 $$f\left(n, {W}_{R}\right)=\frac{{l}_{c\left(n, {W}_{R}\right)}}{{l}_{c\left(n, 0\right)}}=\frac{{\xi }_{c}(n,{W}_{R})}{{\left(4C\right)}^{1/3}} \text{,}$$

where ${l}_{c\left(n, 0\right)}$ is the critical height when the trees have no branches, which is the same as Eq. (1). The quantity ${l}_{c\left(n, {W}_{R}\right)}$ is the critical height when the density distribution parameter is $n$, and the weight ratio is ${W}_{R}$. In other words, Eq. (38) replaces ${\xi }_{c}(n)$ with ${\xi }_{c}(n,{W}_{R})$ in Eq. (37).

We assumed the following models as regression models; each parameter was determined by regression analysis using the non-linear least-squares method. Furthermore, the significance level $\alpha$ was $0.05$:

39 $$\text{Exponential model}:{{P}_{1}e}^{{P}_{2}n} \text{,}$$

40 $$\text{Linear model:} {P}_{3}n+{P}_{4 }\text{,}$$

41 $$\text{Polynomial model:} {P}_{5}{n}^{2}+{P}_{6}n+{P}_{7} \text{,}$$

42 $$\text{Power-law model}:{{P}_{8}n}^{{P}_{9} }\text{.}$$

The exponential model and the power-law model in Eqs. (39) and (42) are simple models expressed in monomial form. The linear and polynomial models in Eqs. (40) and (41) are more complicated than the above models, as they are both expressed in a polynomial form. In particular, the polynomial model in Eq. (41) is a quadratic polynomial and is the most complicated among the four models. The power-law model in Eq. (42) cannot be applied to data with $n\le 0$. Therefore, we use only the data with $n>0$ to perform regression analysis in the case of the power-law model.

## Results and discussion

In this study, based on the physical characteristic values found in^43^ and^44^, we used an elastic modulus $E=1.1\times {10}^{10} \left[\text{N/}{\text{m}}^{2}\right]$, a standard density ${\rho }_{0}=526$ $\left[\text{kg/}{\text{m}}^{3}\right]$, a gravitational acceleration $g=9.81 \left[\text{m/}{\text{s}}^{2}\right]$, and a radius ${r}_{l}=0.23 \left[{\text{m}}\right]$.

### Convergence judgement

**Figure 4:**
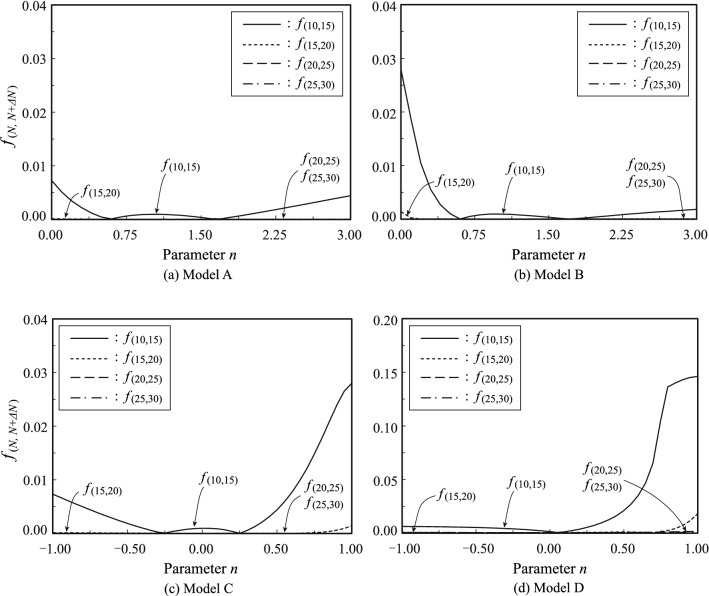
Convergence judgement.

The results of convergence judgement in each model are displayed in Fig. 4. Values for the initial expansion order ${N}_{i}=5$ and the increment $\Delta N=5$. Were used. The values of the convergence judgement function ${f}_{(N,N+\Delta N)}$ are indicated on the vertical axes in the figure and the values of $n$ are indicated on the horizontal axes. In all models, the calculated values converged as the expansion order $N$ increased. However, the convergence of Model D was inferior to the other models that had linear density distributions, i.e., Models A, B, and C. The specific convergence judgement function ${f}_{(\mathrm{20,25})}$ (indicated by the dashed line in each panel) satisfied Eq. (25) in Models A, B, and C. In contrast, this function did not satisfy Eq. (25) in Model D. The specific convergence judgement function ${f}_{(\mathrm{25,30})}$ (represented by the single-dotted line in each panel) satisfied Eq. (25) for all the models. Therefore, we selected $N=25$ as the expansion order.

### Effect of the density distribution on the critical height

**Figure 5:**
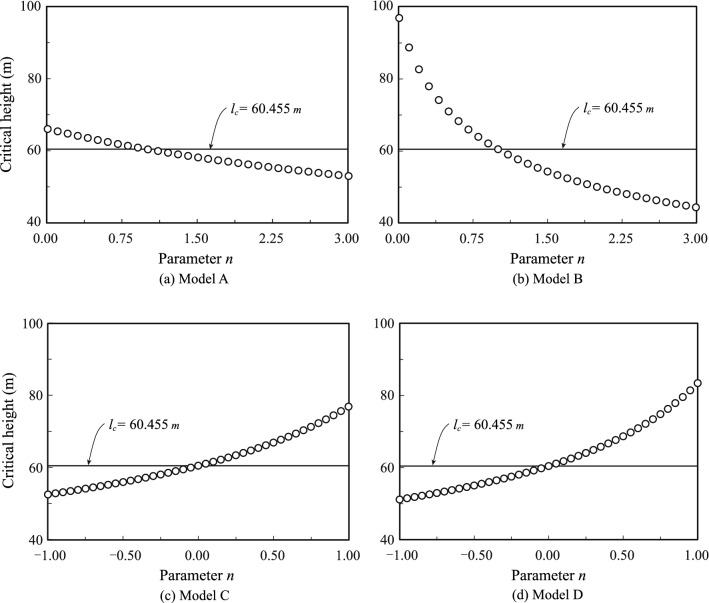
Relationship between critical height ${l}_{c}$ and parameter $n.$

The relationship between the critical height ${l}_{c}$ and the density distribution parameter $n$ is illustrated in Fig. 5. The values of the critical height ${l}_{c}$ are indicated on the vertical axes in the figure, and the values of $n$ are indicated on the horizontal axes.

In Model A, the critical height ${l}_{c}$ slowly decreased with increasing $n$, whereas the critical height ${l}_{c}$ showed a sharp decrease with increasing $n$ in Model B. Comparing the two models in more detail, the critical in Model B was higher than that in model A in the interval $0\le n<1$, whereas the two models showed agreement at $n=1$. In the interval $1<n\le 3$, the critical height in Model A was higher than that in Model B. The reason for these results was that Model A concentrated more weight at the top of the tree in the interval $0\le n<1$, whereas Model B concentrated more weight at the top in the interval $1<n\le 3$. This result indicated that increasing the weight of the upper portion of the tree significantly reduced the critical height, as compared to the result for an increase in the weight of the lower portion.

In Models C and D, which have the same total weight regardless of $n$, the critical height ${l}_{c}$ displayed a curvilinear increase $n$. Retaining the equal total weights, but with the weight distributed such that the upper and lower parts were lighter and heavier, respectively, the critical height showed a dramatic increase. The difference between Model C (straight line) and Model D (curved line) was negligible when $n<0$, the value for Model C being slightly higher. The difference between the two models became apparent at higher values of $n$. The values for Model D were higher than those for Model C when $n>0$*.*

For all models, when the density was constant (in agreement with Greenhill's model), we obtained a value of ${l}_{c}= 60.455 \text{[m]}$, which is almost the same as Greenhill's solution. This indicates that when the governing differential equation is solved by the series solution method, the solutions are as accurate as those obtained by solving exactly if the number of terms $N\ge 25$ is used. The solutions of von Karman and Biot^11^ were derived using $N=6$.

### Derivation a simple scaling law by regression analysis

**Figure 6:**
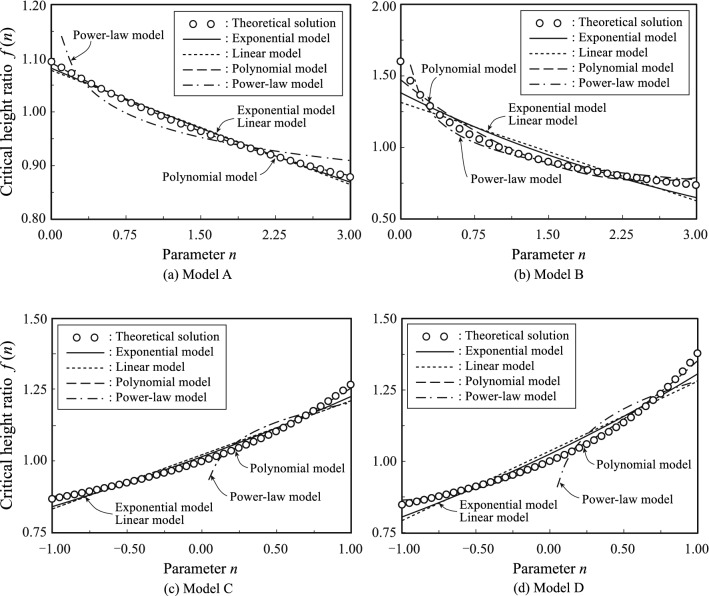
Critical height ratio and regression curve.

The relationship between the critical height ratio $f(n)$ and the density distribution parameter $n$, and the regression curves obtained by non-linear regression analysis are displayed in Fig. 6. The values of the critical height ratio $f(n)$ are indicated on the vertical axes in the figure, and the values of $n$ are indicated on the horizontal axes. Detailed results of the regression analysis are listed in Table 2. All the parameters of all the regression models (${P}_{1}\sim {P}_{9}$) are valid because the p-values are consistently smaller than the significance level $\alpha =0.05$.

**Table 2:** Non-linear regression analysis results.

Regression model	Exponential		Linear		Polynomial			Power-law	
Parameter	${P}_{1}$	${P}_{2}$	${P}_{3}$	${P}_{4}$	${P}_{5}$	${P}_{6}$	${P}_{7}$	${P}_{8}$	${P}_{9}$
**Model A**									
Estimated value	1.081	− 0.073	− 0.071	1.077	0.010	− 1.002	1.091	0.978	− 0.067
p-value	2.0 × 10^–16^	2.0 × 10^–16^	2.0 × 10^–16^	2.0 × 10^–16^	2.0 × 10^–16^	2.0 × 10^–16^	2.0 × 10^–16^	2.0 × 10^–16^	4.2 × 10^–15^
AIC	− 2.311 × 10^2^		− 2.123 × 10^2^		− 3.407 × 10^2^			− 1.431 × 10^2^	
**Model B**									
Estimated value	1.381	− 0.254	− 0.230	1.315	0.109	− 0.556	1.473	0.972	− 0.210
p-value	2.0 × 10^–16^	2.0 × 10^–16^	2.0 × 10^–16^	2.0 × 10^–16^	1.1 × 10^–11^	2.0 × 10^–16^	2.0 × 10^–16^	2.0 × 10^–16^	2.0 × 10^–16^
AIC	− 7.314 × 10^1^		− 5.800 × 10^1^		− 1.078 × 10^2^			− 1.115 × 10^2^	
**Model C**									
Estimated value	1.016	0.190	0.191	1.023	0.068	0.191	0.999	1.207	0.085
p-value	2.0 × 10^–16^	2.0 × 10^–16^	2.0 × 10^–16^	2.0 × 10^–16^	2.0 × 10^–16^	2.0 × 10^–16^	2.0 × 10^–16^	2.0 × 10^–16^	5.6 × 10^–8^
AIC	− 2.154 × 10^2^		− 1.912 × 10^2^		− 3.196 × 10^2^			− 7.314 × 10^1^	
**Model D**									
Estimated value	1.025	0.243	0.245	1.036	0.110	0.245	0.998	1.282	0.115
p-value	2.0 × 10^–16^	2.0 × 10^–16^	2.0 × 10^–16^	2.0 × 10^–16^	2.0 × 10^–16^	2.0 × 10^–16^	2.0 × 10^–16^	2.0 × 10^–16^	1.3 × 10^–7^
AIC	− 1.753 × 10^2^		− 1.513 × 10^2^		− 2.620 × 10^2^			− 5.823 × 10^1^	

In Model A, the critical height ratio (the ratio of the actual critical height ${l}_{c}$ to the value corresponding to the constant-density case) varies from 1.09 to 0.88 (depending on the parameter $n$). Moreover, all regression models, except for the power-law model, are consistent with the theoretical solution. Based on the Akaike Information Criterion (AIC), the polynomial model is the most accurate model. However, for the purpose of obtaining a simple scaling law for trees, the simplicity of the regression model is very important. Therefore, the exponential model is a good regression model because it is superior in terms of both accuracy and simplicity.

In Model B, the critical height ratio varies from 1.60 to 0.73. The polynomial and power-law models are consistent with the theoretical solution. In contrast, the exponential and linear models are not accurate in the neighbourhood of $n=0$ and $n=3$. Notably, the power-law model is not valid at $n=0.$ From the standpoint of AIC, the most accurate model is the power-law model. This model has good accuracy and simplicity.

In Model C, the critical height ratio varies from 1.25 to 0.87. All regression models, except for the power-law model, are consistent with the theoretical solution. The power-law model breaks down when $n<0$, and its error with respect to the theoretical solution is larger than that of the other models. Based on the AIC, the most accurate model is the polynomial model. For the purpose of obtaining a simple scaling law for trees, the exponential model is a good regression model because it is superior in both accuracy and simplicity.

In Model D, the critical height ratio varies from 1.38 to 0.85. Regarding the regression models, all models except for the power-law model are consistent with the theoretical solution. The power-law model cannot account for the critical height when $n<0$, and its error with respect to the theoretical solution is larger than that of the other models. The exponential model shows good accuracy and simplicity.

To derive a simple scaling law such as Greenhill's, we considered the use of simple fractions to express the critical heights. Simple expressions of the regression models considered optimal for each density model in terms of appropriate fractions are shown in Table 3. The limits of application for each formula are also listed in the table.

**Table 3:** Simplified formulas for critical height.

Model	Formula	Applicable limit
A	${l}_{c}={\frac{11}{10}{e}^{-3n/40}\left(C\frac{E}{\gamma }{r}_{l}^{2}\right)}^{1/3}$	$0\le n\le 3$
B	${l}_{c}={{n}^{-1/5}\left(C\frac{E}{\gamma }{r}_{l}^{2}\right)}^{1/3}$	$0<n\le 3$
C	${l}_{c}={{e}^{n/5}\left(C\frac{E}{\gamma }{r}_{l}^{2}\right)}^{1/3}$	$-1\le n\le 1$
D	${l}_{c}={{e}^{n/4}\left(C\frac{E}{\gamma }{r}_{l}^{2}\right)}^{1/3}$	$-1\le n\le 1$

### Application to real trees

**Figure 7:**
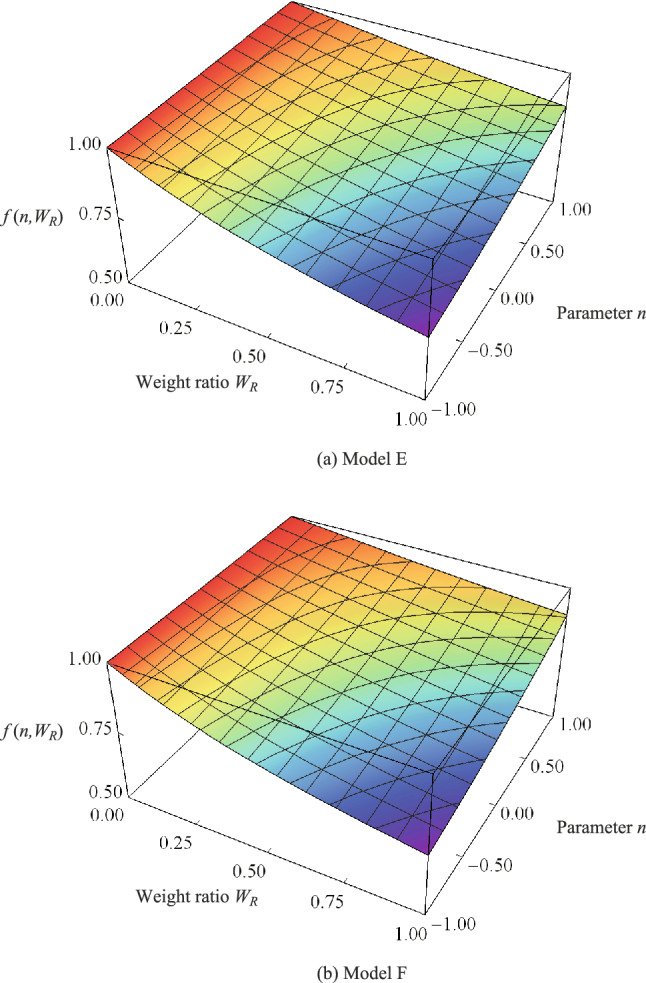
Relationship between the critical height and the branch distribution (contained in the weight ratio).

The results of the application to a real tree, in which the balance of branch and trunk weights and their distribution on the critical height were investigated, are displayed in Fig. 7. The results for Models E and F are shown in panels (a) and (b), respectively.

In both models, the critical height increased with increasing $n$. Focusing on ${W}_{R}$, it was possible to increase the critical height by making the top lighter, even when the same number of branches and leaves were used. Comparing Models E and F, the quadratic distribution was more favourable when the top was lighter, and the linear distribution was more favourable when the top was heavier.

Based on King et al.’s study of the weight ratio^40^ and Hirata’s study of the weight distribution of trees^41^, the value ranges we used for ${W}_{R}$ and $n$ for real trees were: $0.1\le {W}_{R}\le 0.6$ and $0\le n\le 1$. We found the following maximum and minimum values of $f\left(n,{W}_{R}\right)$ in these ranges:

43 $$\text{Model E}: {f}_{max}=0.985\text{, } { f}_{min}=0.855 \text{,}$$

44 $$\text{Model F}: {f}_{max}=0.988\text{, } { f}_{min}=0.855 .$$

The physical meaning of the quantities ${f}_{max}$ and ${f}_{min}$ in Eqs. (43) and (44) may be expressed as "the maximum and minimum values of the critical height ratio with respect to the case when all branches and leaves are cut off". Even if a real tree has the most unfavourable configuration ($n=0, {W}_{R}=0.6$), the mechanical critical height is reduced by only about 15%.

Niklas reported that the critical height ${l}_{c}$ obtained by the Greenhill equation has a safety factor of approximately $S=4.0$ ($S={l}_{real}/{l}_{c}$) with respect to the actual height of the tree, ${l}_{real}$^45^. Combining the results of Eqs. (43) and (44) with the results of Niklas et al., we can estimate that the safety factor for the critical height equation, considering the weight distribution of trees and the weight ratio of the trunk to the branches, lies approximately in the following range:

45 $$3.4\le S\le 3.9 .$$

Actual trees have highly diverse weight distributions. However, by performing calculations using a density distribution function that can represent such a wide range of weight distributions and then refining the results using actual measurement data, it is possible to estimate the critical height more accurately than using the critical height equation which ignores existing branch and leaf weights.

### Buckling modes under self-weight

**Figure 8:**
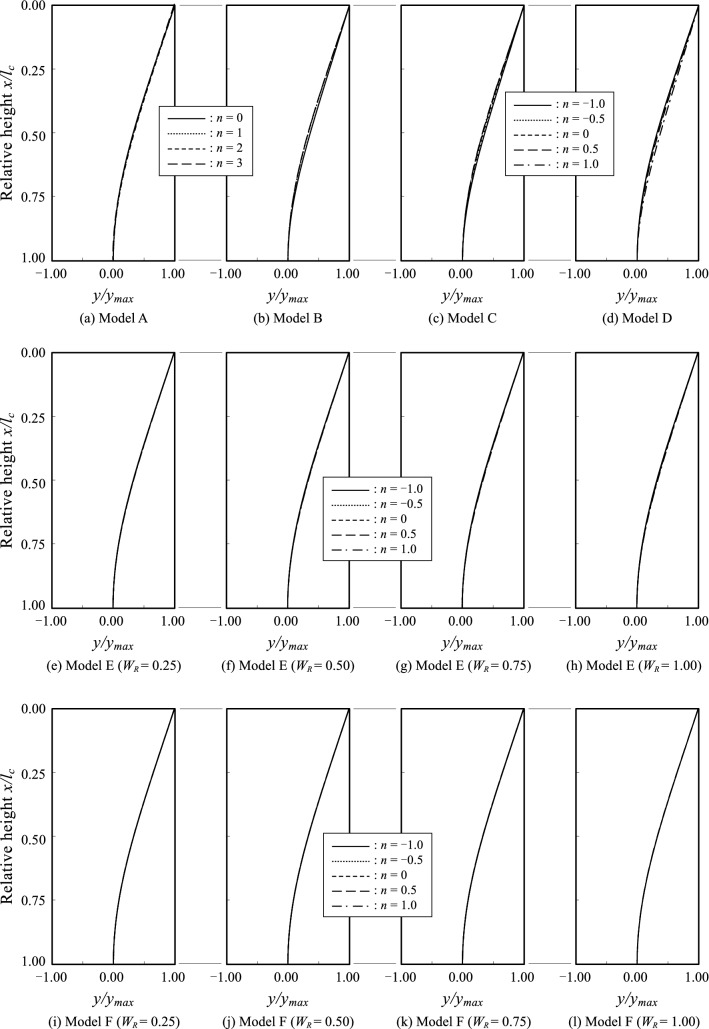
Buckling modes.

The buckling modes under self-weight for all the density models are represented in Fig. 8. The vertical axis in each panel represents the dimensionless coordinate $x/{l}_{c}$, and the horizontal axis in each panel represents the dimensionless deflection $y/{y}_{max}$. The buckling modes for $n=0, 1, 2~{\text{and}}~3$ are shown for models A and B. The buckling modes for $n=-1, -0.5, 0, 0.5~\mathrm{ and }~1$ are shown for models C, D, E and F.

There were no significant differences in the buckling modes for any of the density models. The results are similar to those of the first-order mode in Euler buckling, where the deflection uniformly increases from the fixed end to the free end. No significant difference in the mode shapes was observed when the density distribution parameters $n$ and the weight ratios ${W}_{R}$ were changed.

## Conclusions

In this study, to clarify the effect of the weight distribution of trees on their critical height, the critical height for self-weight buckling was formulated for cylindrical models with various weight distributions and the critical height equations that include the influence of branch weight was derived for the first time. Regression analysis was performed on the discrete theoretical solutions, and a simple relationship between the weight distribution and the limit height was derived for each density model. Furthermore, using the obtained critical heights, the mode shapes under buckling under self-weight for various weight distributions were obtained. As a result, the following new findings were obtained:

1. When a certain number of branches and leaves are distributed in the height direction, the risk of buckling due to self-weight can be reduced if the upper part of the trees is lighter and the lower part is heavier. This method of weight distribution can increase the critical height by a maximum of approximately 1.25 times for a linear distribution and by a maximum of approximately 1.38 times for a curvilinear distribution, compared with a constant weight distribution in the vertical direction. In addition, the total allowable weight is approximately 2 times larger in the case of a linear distribution and approximately 2.6 times larger in the case of a curved distribution.
2. The buckling mode when self-weight buckling occurs is similar to the first buckling mode in the case of general long columns, regardless of the weight distribution in the height direction. In addition, the mode shape hardly changes even if the distribution form changes in the same density model.
3. Based on the weight distributions of real trees and the measurements of branch weights, it can be said that trees distribute their branches and leaves in a very rational way that has little effect on the critical height. Even if we consider the most unfavourable condition of a real tree ($n=0, {W}_{R}=0.6$), the critical height is only reduced by approximately 15% from the condition with no branches or leaves at all. Based on the findings of Niklas^45^, it can be estimated that the safety factor of the actual trees is approximately 3.4 to 3.9 in relation to the theoretical critical height of the present study, considering the branches. The method in this study can obtain more accurate critical height than previous methods that ignore branch weight because a safety factor of approximately 4 was reported in previous research^45^.

In future work, based on the methods and findings of this study, the shape law and rationality of plant morphology will be further explored from a theoretical point of view. Currently, we plan to investigate the effects of crown weight and position using more complex density function models and to extend the method to hollow cylindrical models such as bamboo and to solid and hollow models with a taper. In the future, we intend to summarise these findings and propose the next generation of material-saving and high-performance structural designs and rational new design methods.
